# Organophosphorus Reinforced Poly(vinyl alcohol) Nanocomposites Doped with Silver-Loaded Zeolite L Nanoparticles as Sustainable Materials for Packaging Applications

**DOI:** 10.3390/polym15112573

**Published:** 2023-06-04

**Authors:** Tăchiță Vlad-Bubulac, Corneliu Hamciuc, Diana Serbezeanu, Dana Mihaela Suflet, Daniela Rusu, Gabriela Lisa, Ion Anghel, Dana-Maria Preda, Totka Todorova, Cristina Mihaela Rîmbu

**Affiliations:** 1 “Petru Poni” Institute of Macromolecular Chemistry, 41A Grigore Ghica Voda Alley, 700487 Iasi, Romania; chamciuc@icmpp.ro (C.H.); diana.serbezeanu@icmpp.ro (D.S.); dsuflet@icmpp.ro (D.M.S.); rusu.daniela@icmpp.ro (D.R.); 2 Department of Chemical Engineering, Faculty of Chemical Engineering and Environmental Protection, “Gheorghe Asachi” Technical University of Iasi, 73 Bd. Mangeron, 700050 Iasi, Romania; gabriela.lisa@academic.tuiasi.ro; 3 Fire Officers Faculty, Police Academy “Alexandru Ioan Cuza”, Morarilor Str. 3, Sector 2, 022451 Bucharest, Romania; ion.anghel@academiadepolitie.ro (I.A.); mariadana523@yahoo.com.sg (D.-M.P.); 4 Institute of Catalysis, Bulgarian Academy of Sciences, Acad. G. Bonchev St., bl.11, 1113 Sofia, Bulgaria; t.todorova@ic.bas.bg; 5 Department of Public Health, Iasi University of Life Sciences, 8 Sadoveanu Alley, 707027 Iasi, Romania; crimbu@yahoo.com

**Keywords:** sustainable development, poly (vinyl alcohol), organophosphorus flame retardant, silver-loaded zeolite, thermal stability, antimicrobial activity

## Abstract

The sustainable development of innovative eco-friendly multifunctional nanocomposites, possessing superior characteristics, is a noteworthy topic. Novel semi-interpenetrated nanocomposite films based on poly(vinyl alcohol) covalently and thermally crosslinked with oxalic acid (OA), reinforced with a novel organophosphorus flame retardant (PFR-4) derived from co-polycondensation in solution reaction of equimolar amounts of co-monomers, namely, bis((6-oxido-6H-dibenz[c,e][1,2]oxaphosphorinyl)-(4-hydroxyaniline)-methylene)-1,4-phenylene, bisphenol S, and phenylphosphonic dichloride, in a molar ratio of 1:1:2, and additionally doped with silver-loaded zeolite L nanoparticles (ze-Ag), have been prepared by casting from solution technique. The morphology of the as prepared PVA-oxalic acid films and their semi-interpenetrated nanocomposites with PFR-4 and ze-Ag was investigated by scanning electron microscopy (SEM), while the homogeneous distribution of the organophosphorus compound and nanoparticles within the nanocomposite films has been introspected by means of energy dispersive X-ray spectroscopy (EDX). It was established that composites with a very low phosphorus content had noticeably improved flame retardancy. The peak of the heat release rate was reduced up to 55%, depending on the content of the flame-retardant additive and the doping ze-Ag nanoparticles introduced into the PVA/OA matrix. The ultimate tensile strength and elastic modulus increased significantly in the reinforced nanocomposites. Considerably increased antimicrobial activity was revealed in the case of the samples containing silver-loaded zeolite L nanoparticles.

## 1. Introduction

Nowadays the continuously increasing production of single-use plastics appealing for packaging applications represents a significant environmental problem since the single-use plastic industry is actually manufacturing more petroleum-based plastic materials than ever before on a global scale [1,2,3]. With more than 139 million tons of disposable plastic waste generated and released directly into the environment around the world in 2021, coordinated efforts are to be made to prevent, minimize, or manage plastic waste [4,5]. Regardless of increasing end-users awareness, rigorous regulation, and policymakers’ or stakeholders’ consideration, the most optimistic predictions estimate for the next three years an increase of 30% in disposable plastic waste, which are usually ”handled” by controlled burning (35%), burying in managed landfills (31%) and, even worse, by direct throwing away on land or into the ocean [5,6]. As a consequence, restraining contamination, relieving the global warming crisis, settling worldwide ecosystems, and conserving biodiversity, while enhancing food security and improving human health and quality of life are facing major challenges and need to be urgently addressed and harmonized. In this context, sustainable packaging, which refers to keywords such as green, reusable, or eco-friendly packaging, plays a pivotal role in reducing pollution and waste [7]. 

In order to be suitable for sustainable packaging any material must be safe for both the environment and humans [8]. On one hand, biopolymers are the material of primary choice for environmentally friendly packaging because they outperform synthetic polymers in many ways, in terms of biodegradability, non-toxicity, non-health concern, bioactivity, abundance on a large scale, and suitability for the expected increasing demand on the market (80% of European consumers are eager to purchase products with little to no negative environmental impact) [9,10,11]. On the other hand, bio-based polymers are less economical, typically costing two to three times as much as the most popular conventional plastics such as PE or PET [12]. Other disadvantages, such as weaker mechanical and thermomechanical properties, shorter lifetime, advanced water vapour permeability, or even the concern of interference with the food supply chains, in the case of biopolymers based on terrestrial crops, are worthy to be mentioned [13,14]. To meet the halfway of the requirements vs. performances balance, the second option is represented by semisynthetic polymers and their composites either with various biobased plastics or with various additives or fillers specifically introduced to bring additional functionality to the desired material [15,16,17]. 

Due to its outstanding performances, such as the ability to be processed in various forms with 2D or 3D spatiality (films [18], multilayer coatings [19], membranes [20], nanofibers [21,22], hydrogels [23], xerogels [24], etc.), biodegradability, biocompatibility, eco-friendly characteristics [25], oxygen barrier properties, good chemical resistance, transparency in the visible range and acceptable thermomechanical properties, poly(vinyl alcohol), PVA, is quoted exponentially as one of the most widely used water-soluble polymers appealing for packaging industry [26,27,28,29,30,31]. Although PVA has some limitations such as poor ultraviolet (UV) and moisture barrier properties, high flammability, and weak thermal stability, yet, it presents remarkable blending ability, good interfacial adhesion, and attractive compostable capacity [32,33]. As sustainable material for rigid or flexible packaging sectors, the main drawbacks of PVA are related to its high-water absorption capacity and lack of antioxidant and antimicrobial properties. The literature survey reveals that many groups have addressed the aforementioned disadvantages in great detail, with each drawback being partially remedied by judicious reinforcement of the PVA matrix in conjunction with suitable biopolymers and/or with well-known additives bearing specific improvements. Yet, there is still room for sustainable development via simultaneous combination of biodegradable semi-synthetic polymers with exceptional additives, or doping nanoparticles which may improve certain synergistic functionalities in the targeted material. Modulating the properties of PVA with organophosphorus compounds and with silver-containing additives, while semi-interpenetration networking with low-molecular dicarboxilic acids, is the key to simultaneously improvement of the thermal stability, resistance against fire and moisture and imparting intrinsic antimicrobial properties. Thus, the present paper focuses on the synthesis and characterization of a series of PVA composites reinforced with oxalic acid, which was chosen to controthe l water solubility of the hydrophilic PVA chains by physical and chemical crosslinking. A novel organophosphorus flame retardant (PFR-4) derived from co-polycondensation in solution reaction of echimolar amounts of co-monomers, namely, bis((6-oxido-6H-dibenz[c,e][1,2]oxaphosphorinyl)-(4-hydroxyaniline)-methylene)-1,4-phenylene, bisphenol S, and phenylphosphonic dichloride, in a molar ratio of 1:1:2, was utilized to impart thermal stablility and resistance against flame and fire, while doping with silver-loaded zeolite L nanoparticles (ze-Ag) was expected to activate antimicrobial properties against different microbial cultures.

## 2. Materials and Methods

### 2.1. Materials

Partially hydrolyzed PVA—Mowiol 26-88 (Kuraray Poval^TM^) with a molecular weight of 160,000 g/mol and a degree of hydrolysis of 87.7% was purchased from Zauba (Munchen, Germany) and it was used as received to manufacture the PVA/OA reinforced films. Oxalic acid, terephthalaldehyde, 4-aminophenol, AgNO_3_, 4,4′-sulfonyldiphenol, phenylphosphonic dichloride, *N*-methyl-2-pyrrolidone (NMP), *N,N*-dimethylformamide (DMF) were provided from Sigma-Aldrich, Steinheim, Germany and used without further purification. 9,10-Dihydro-9-oxa-10-phosphaphenanthrene-10-oxide (DOPO) was purchased from Chemos GmbH, Altdorf, Germany, and dehydrated before use. Following a previously described procedure, 4,4′-terephthalylidene-bis(p-hydroxyaniline), was prepared from 4-aminophenol and terephthalaldehyde [34] and then, by addition of DOPO, according to the method previously reported [35], bis((6-oxido-6H-dibenz[c,e][1,2]oxaphosphorinyl)-(4-hydroxyaniline)-methylene)-1,4-phenylene has been synthesized. Dispersed extra pure silicon dioxide was purchased from Merck, Gernsheim, Germany, aluminium 99.97% from Acros Organics, Geel, Belgium, NaOH pellets 98%, and KOH pellets 85% from Sigma Aldrich, Steinheim, Germany. Silver-loaded zeolite L nanoparticles (ze-Ag) have been prepared according to a method described in detail in our previous paper [36]. All other materials used in the experiments were acquired from commercial sources and were used without further purification.

### 2.2. Preparation of Oligophosphonate, PFR-4

PFR-4 was obtained by solution polycondensation reaction of an equimolar amount of DOPO-containing bisphenol, namely bis((6-oxido-6H-dibenz[c,e][1,2]oxaphosphorinyl)-(4-hydroxyaniline)-methylene)-1,4-phenylene and 4,4′-sulfonyldiphenol with phenylphosphonic dichloride according to an updated procedure described in a previous paper [35]. In summary, in a 100 mL round flask equipped with a reflux condenser, magnetical stirrer and nitrogen inlet and outlet, bis((6-oxido-6H-dibenz[c,e][1,2]oxaphosphorinyl)-(4-hydroxyaniline)-methylene)-1,4-phenylene (7.48 g, 0.01 mol), bisphenol S (2.5 g, 0.01 mol), N-methyl-2-pyrrolidone (NMP) (50 mL) and triethylamine (3 mL) were introduced and mixed under vigorous stirring. After a homogeneous solution was obtained, phenylphosphonic dichloride (3.9 g, 0.02 mol) was introduced carefully under stirring, during a period of 60 min. The reaction mixture was allowed to react over 8 h on heating the content to 50 °C. The resulting mixture was then brought to room temperature before being added to the methanol. Filtering and re-dissolving in NMP were done before a dark brownish-red powdery solid (yield: 90%) was produced after the oligomer was separated by precipitation in water, repeated water washes, and 24 h of drying at 60 °C in a vacuum oven.

FTIR (KBr, cm^−1^): 3292 (NH), 3065 (C–H aromatic), 1475 (P–Ar), 1210 and 1140 (P=O), 1045 (P–O–C), 925 (P–O–Ar).
^1^H-NMR (400 MHz, DMSO-*d6*, δ, ppm): 8.52 (d, 2H), 8.18 (m, 4H), 8.02 (d, 4H), 8–7.5 (m, 6H), 7.99 (d, 4H), 7.5–7.05 (m, 18H), 6.52 (m, 8H), 6.1 and 5.7 (m, 2H, N–H), 5.4 and 4.85 (m, 2H, CH–P).

### 2.3. Preparation of PVA/OA Films

**Figure 1:**
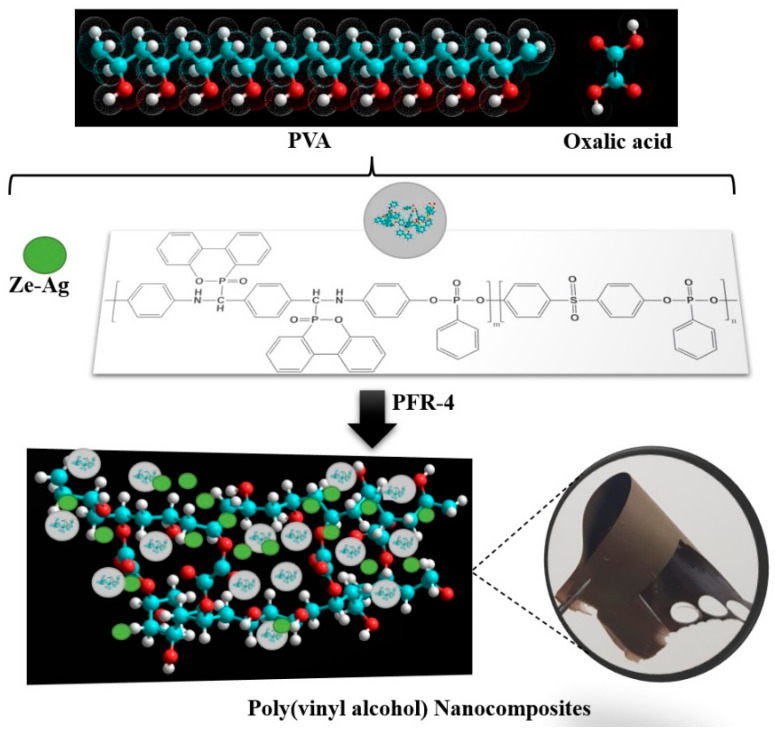
Graphical representation of the reinforced PVA/OA/PFR-4/ze-Ag composites.

In the first step, the corresponding amounts of PVA and oxalic acid (15% wt.), as listed in Table 1, were dissolved in distilled water to obtain solutions of 10% concentration. Following this procedure, the second solutions/suspensions were obtained by dissolving/dispersing PFR-4 and ze-Ag additives in a small volume of DMF (1.8 to 3.6 mL). These two solutions were then mixed, stirred vigorously, and ultrasonicated for 30 min before pouring these homogeneous composite mixtures into 10 × 10 cm^2^ Teflon molds and slowly evaporating the solvents in ambient conditions overnight. Subsequently, in the last step of the manufacturing procedure, the molds were placed in an oven and an advanced drying treatment was applied by heating the samples under vacuum for a period of 12 h at 55 °C to obtain freestanding, flexible films, as shown in the inset in Figure 1. The complete data reporting the synthesis of the film composites are presented in Table 1 while a schematic representation of the process of preparation of the PVA-OA composites is presented in Figure 1.

### 2.4. Methods

#### 2.4.1. FTIR Spectra

A LUMOS Microscope Fourier Transform Infrared (FTIR) spectrophotometer (Bruker Optik GmbH, Ettlingen, Germany), equipped with an attenuated total reflection (ATR) device was used to record the scans between 4000 and 500 cm^−1^ at a resolution of 4 cm^−1^.

#### 2.4.2. Scanning Electron Microscopy

Scanning electron microscopy (SEM) was performed on a Verios G4 UC Scanning Electron Microscope (Thermo Scientific, Brno, Czech Republic). The samples were coated before examination with 6 nm platinum using a Leica EM ACE200 Sputter coater in order to increase electrical conductivity and reduce charge buildup which can occur during exposure to the electron beam. SEM investigations were performed in High Vacuum mode using a detector for high-resolution images (Through Lens Detector, TLD, Jeol, Freising, Germania) at an accelerating voltage of 5 kV, the magnification being indicated on the micrographs. The Verios G4 UC microscope is equipped with an X-ray detection system (Energy Dispersive X-ray Spectroscopy analyzer—EDX) for qualitative elemental composition and map distribution of the elements in the sample.

#### 2.4.3. Contact Angle

The wettability of the surfaces was evaluated by static contact angle measurements using the sessile drop method at room temperature. The measurements were carried out using a CAM 101 Optical Contact Angle Instrument (KSV Instruments, Helsinki, Finland) equipped with a special optical system connected to a computer. ~1 μL of tested liquids (water (W) and ethylene glycol (EG)) were placed with a Hamilton syringe on the surface s and the images were sent via the CCD camera to the computer for analysis. Ten photos were recorded at an interval of 0.016 s. All the measurements were done in triplicate and the results were recorded as mean ± standard deviation. The surface parameters (adhesion work, surface free energy, interfacial solid-liquid tension) were estimated based on contact angle values and the following equations developed by Owens-Wendt-Rabel-Kaelbe [37]:

(1) $$W_{a}=\gamma_{LV}+\gamma_{SV}-\gamma_{SL}=\gamma_{LV}\left(1+\cos\theta \right)$$

(2) $$\gamma_{SV}=\gamma_{SV}^{p}+\gamma_{SV}^{d}$$

(3) $$\gamma_{LV}\left(1+\cos\theta \right)=2\sqrt{\gamma_{SV}^{p}\gamma_{LV}^{p}}+2\sqrt{\gamma_{SV}^{d}\gamma_{LV}^{d}}$$

(4) $$\gamma_{SL}=\gamma_{SV}+\gamma_{LV}-2\left(\sqrt{\gamma_{SV}^{p}\gamma_{LV}^{p}}+\sqrt{\gamma_{SV}^{d}\gamma_{LV}^{d}}\right)$$

where, *W*_a_ is the adhesion work, γ_LV_ is the liquid-vapor surface tension, γ_SV_ is the energy of the surface, γ_SL_ is the solid drop interfacial tension, cos θ is the drop—surface contact angle, γ^p^_SV_ is the polar component of the surface tension that including two types of coulomb interactions dipole-dipole and dipole-induced dipole and γ^d^_SV_ is the dispersive component of the surface tension which represent van der Waals interactions. The surface tension values for the two liquids and their components (mN/m) are γ_LV_ = 72.8, γ^p^_LV_ = 51, γ^d^_LV_ = 21.8 for water and γ_LV_ = 48, γ^p^_LV_ = 19, γ^d^_LV_ = 29 for ethylene glycol, respectively.

#### 2.4.4. Differential Scanning Calorimetry (DSC) Measurements

Differential scanning calorimetry (DSC) measurements of composite films were carried out using a Mettler Toledo DSC1 type device in an inert atmosphere, with a heating rate of 10 °C/min. The glass transition temperature (T_g_) was considered as the midpoint of the inflection tangent, at the second heating scan in the temperature range 25–140 °C.

#### 2.4.5. TGA Measurements

The thermogravimetric (TG) and derivative thermogravimetric (DTG) curves were processed with Mettler Toledo STARe software (Version 9.10 (Giessen, Germany)). The reproducibility of the TG/DTG curves was verified by recording several tests for the investigated sample using the same conditions and differences of less than 1% were found. TG and DTG curves were recorded in a nitrogen atmosphere with a heating rate of 10 °C/min in the temperature range of 25–750 °C.

#### 2.4.6. Microscale Combustion Calorimetry (MCC)

The flammability of samples in controlled temperature circumstances was evaluated using microscale combustion calorimetry (MCC) experiments; the temperature in the combustor was 900 °C, and the pyrolyzer was heated up to 750 °C at a rate of 1 °C/s. The tests were carried out in accordance with “Method A.” (ASTM D7309-13) [36].

#### 2.4.7. Mechanical Testing

The tensile strength of the films was evaluated using a TA.XT Plus Texture Analyzer, at room temperature. Test samples, rectangular in shape (50 mm/10 mm length) were kept for 1 h in a desiccator with 75% humidity (NaCl-H_2_O). The testing samples were fixed between two clamps that were positioned at a 20 mm distance. The film’s thickness was approximately 0.16 mm and the speed of the tensile measurements was 0.5 mm/s at 25 °C. The elastic modulus (Young’s modulus) was calculated from the slope of stress–strain curves between 1 and 2% elongation. The tests were performed in triplicate, and the mean value was calculated and noted.

#### 2.4.8. Antimicrobial Activity

Antimicrobial activity testing is performed under specific conditions for in vitro biological assays by contacting some samples with different microbial cultures. The methods are selected and sometimes adapted according to the structure and nature of the samples to be tested. The essence of the method consists in the ability of some antimicrobial molecules to be released and diffused in liquid or solid culture media in which microorganisms are cultured, with which they can interact and against which they can have an inhibitory or microbicidal effect.

The antimicrobial activity of PVA/OA films (PVA/OA-0, PVA/OA-1, PVA/OA-2, PVA/OA-3, PVA/OA-4) was tested using the Kirby-Bauer diffusion method. Standardized bacterial cultures of *Staphylococcus aureus* ATCC 25923, *Methicillin-resistant Staphylococcus aureus* (MRSA) ATCC 33591 (Gram-positive), *Escherichia coli* ATCC 25922, were prepared to a cell density corresponding to 0.5 McFarland turbidity standard (1.5 × 10^8^ CFU/mL). One mL of each microbial suspension was distributed into Petri dishes (90 mm) over which a Mueller-Hinton agar (Oxoid) culture medium was spread.

Films (10 mm diameter disks) were distributed on the surface of the culture media. The plates were prepared in triplicate and incubated at 37 °C for 24 h. Antimicrobial activity was evaluated by measuring the width of the zone of inhibition formed around the PVA/OA sample.

To analyze the results, two standard descriptive methods were used: calculating the sample mean and standard deviation (SD). The mean was obtained by adding up all the data points and dividing by the number of tests done in triplicate. Standard deviation is a fundamental statistical analysis tool that represents the positive square root of the variance. It measures the amount of variation or dispersion in the dataset. A low value for standard deviation implies that the values are closely clustered around the mean, while a high value indicates that they are more spread out from the mean.

## 3. Results

### 3.1. Structural Investigation

**Figure 2:**
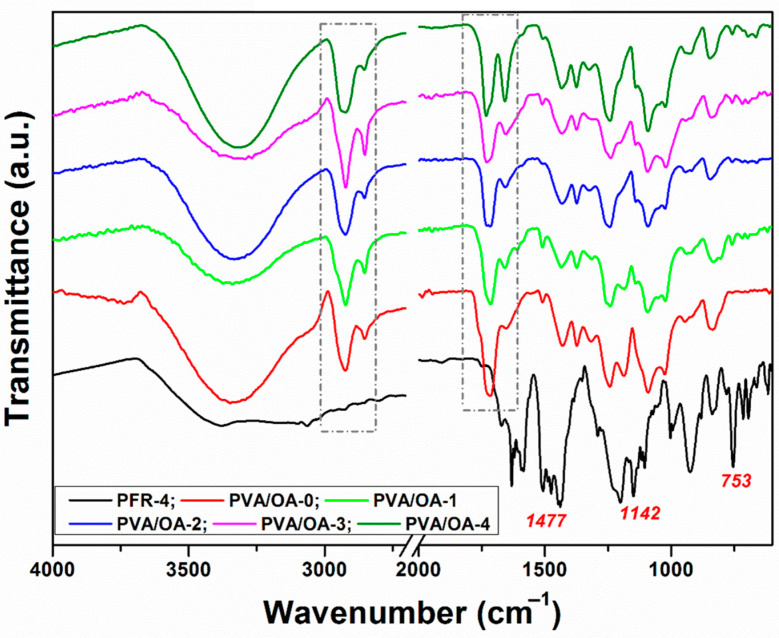
FTIR spectra of PFR-4, PVA/OA-0, and reinforced PVA/OA (1–4) composites.

The FTIR spectra of PFR-4, PVA/OA-0, and PVA/OA (1–4) were presented in Figure 2.

All samples PVA/OA-0-PVA/OA-4 showed the characteristic bands for polyvinyl alcohol centered at ~3300 cm^−1^ attributed to the ν_O-H_ vibration, at 2921 cm^−1^ and 2854 cm^−1^ (dashed rectangle in Figure 2) which are assigned to the ν_C-Hsim_ and ν_C-Hasim_ bands, respectively. The vibrations at 1725 cm^−1^ and 1651 cm^−1^ (dashed rectangle in Figure 2) are attributed to the ν_C = O_ and ν_C = C_ bands, respectively. The bands at 1410 cm^−1^ and 1376 cm^−1^ are due to δ_C-H_ and ω_C-H_ vibrations. The vibration at 1245 cm^−1^ is attributed to ω_C-H_ and the ν_C-O_ vibration is present at 1088 cm^−1^ and 1021 cm^−1^. The vibration at 842 cm^−1^ is attributed to ρ_CH2_ [38]. The absorption bands around 1477, 1218, and 1142 cm^−1^ were attributed to the stretching vibration of the P–Ar, P–O, and P–O–C bonds, respectively. In the FTIR spectra of PVA/OA-0-PVA/OA-4 films, there is a diminished peak near 753 cm^−1^, corresponding to P–O–Ph, which in PFR-4 flame retardant appears as a sharp peak. However, with the addition of 0.13 g and 0.26 g of ze-Ag into the PVA/OA-1 film, the same vibration stretch was weakened. The absence of a new peak with the introduction of ze-Ag indicates no chemical reaction or some hydrogen bonding, all this information indicating that between PVA/OA film and ze-Ag implied rather just physical blending. In the region of 1024–956 cm^−1^, characteristic absorbtion bands related to asymmetric stretching of Si–O and stretching vibration of Al–O bond, belonging to SiO_4_ and tetrahedral AlO_4_ in PVA/OA-3 and PVA/OA-4 are suggestive for the presence of ze-Ag into the composites even if, as it can be seen, the strong band located at 925 cm^−1^ (P–O–Ar) coming from PFR-4 is responsible for the masking of the accurate appearance of the above mentioned characteristic absorption bands.

### 3.2. Morphology Investigation

**Figure 3:**
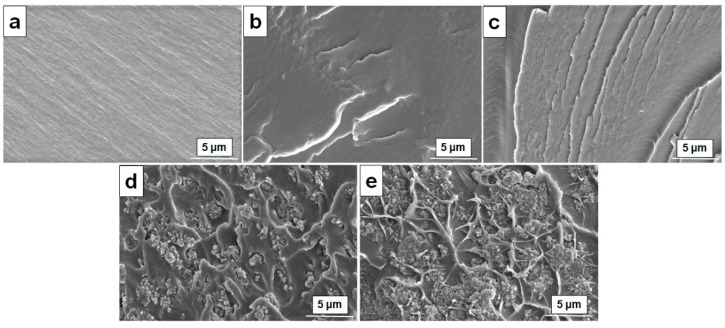
SEM micrographs of the PVA/OA-0 (**a**), PVA/OA-1 (**b**), PVA/OA-2 (**c**), PVA/OA-3 (**d**), and PVA/OA-4 (**e**).

The PVA/OA-0, PVA/OA-1, and PVA/OA-2 exhibited a homogenous surface, without defects or voids. Also, from the SEM images of the PVA/OA-1 and PVA/OA-2 (Figure 3b,c) can be observed the uniform distribution of the PFR-4 into PVA/OA-0 film. In the case of the PVA/OA-3 and PVA/OA-4, it can be observed some agglomeration of ze-Ag particles. The addition of ze-Ag has changed the microstructure of the PVA/OA film (Figure 3d,e). From Figure 3d,e can be observed that the addition of the ze-Ag to the PVA/OA film leads to a rough surface and spherical particles. Also, the cross-sectional micrographs of the PVA/OA-3 and PVA/OA-4, respectively, present a smooth morphology, and the particle (zeolite) density increases with increasing zeolite content in the matrix (Figure 3d,e). This means that the ze-Ag was well mixed into PVA/OA film. It should also be noted that no cracks were observed between the zeolite particles and the PVA membrane.

**Figure 4:**
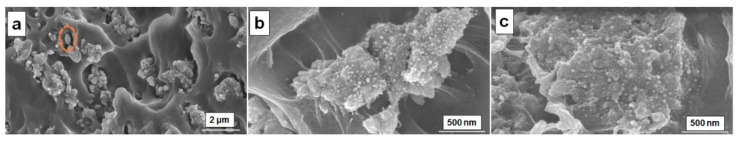
SEM micrographs of the PVA/OA-5 (**a**), PVA/OA-4 (**b**), and PVA/OA-5 (**c**).

Also, SEM confirmed the characteristic morphology of the ze-Ag (Figure 4a, marked circle) loaded into PVA/OA films. In the Figure 4b,c, the observed small dots confirmed the presence of silver around the zeolite particles. Additionally, the distribution of the ze-Ag can be found as uniform without any notable agglomeration under the observation conditions.

**Figure 5:**
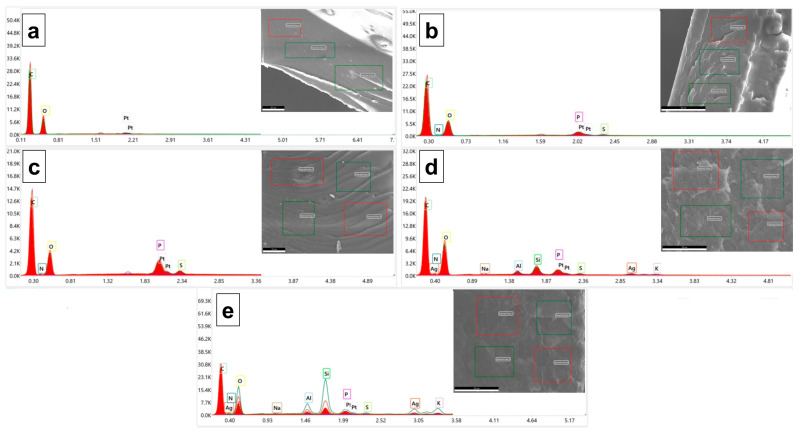
EDX spectra of the PVA/OA-0 (**a**), PVA/OA-1 (**b**), PVA/OA-2 (**c**), PVA/OA-3 (**d**), and PVA/OA-4 (**e**).

The composition of the PVA/OA matrices was evaluated supplementarily by EDX analysis (Figure 5). The elemental analysis revealed a high content of carbon in all the prepared samples. The presence of the P atom on the surface of the PVA/OA (1–4) samples can be observed in Figure 5 (color boxes). The results showed that the P/C ratio increased from 0.00553 ± 3.26 * 10^−5^ (PVA/OA-1) to 0.01487 ± 0.0010 (PVA/OA-2) when the amount of PFR-4 was increased from 0.2 g to 0.4 g into the PVA/OA matrix. For the samples PVA/OA-3 and PVA/OA-4, respectively, it was calculated the metal ratio taking Si as the internal standard. The results showed that the metal ratio is relatively low when expressed in term of the Ag/Si ratio and also can be observed an increase of the Ag/Si ratio when the amount of ze-Ag increased from 0.13 g (0.1187 ± 0.010) to 0.26 g (0.1555 ± 0.043). Strong signal characteristics to the silver atoms in the samples PVA/OA-3 and POVA/OA-4 can be observed at the 3 KeV confirming the presence of the AgNP in the PVA/OA matrices [39].

**Figure 6:**
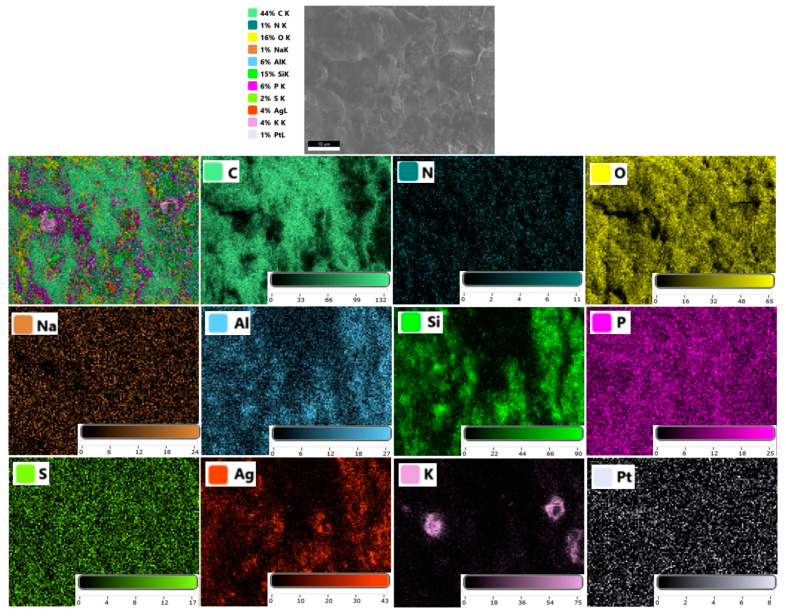
EDX elemental mapping of the reinforced PVA/OA-4 composite film.

Also, the distribution of the different elements of the PVA/OA-4 matrices has been further studied by SEM/EDX mapping analysis. According to the EDX spectra and mapping patterns, the randomly distribution of the ze-Ag nanoparticles into the PVA/OA-4 was observed (Figure 6).

### 3.3. Surface Characteristics

The surface of the PVA/OA films was analyzed from the energetic point of view using the method of determining the contact angle. In this case, two different liquids were used to determine the contact angle, namely distilled water and ethylene glycol. The values obtained for determining the contact angle for the PVA/OA films are presented in Table 2. 

Based on this information it was possible to calculate the surface tension (γ_LV_ (surface tension of the liquid in equilibrium with saturated vapors), γ_SV_ (surface tension of the solid in equilibrium with saturated vapors), γ_SL_ (interfacial tension between solid and liquid)), adhesion work and the polarity (Table 3). As shown in the Table 2, the contact angle for PVA/OA-0 was measured at 131.49° ± 0.28°. The contact angle slightly decreased to 99.41° ± 1.02° for PVA/OA-1, 114.63° ± 1.95° for PVA/OA-2, 113.04° ± 0.92° for PVA/OA-3 and 124.66° ± 0.87° for PVA/OA-4. The lowest values were observed for PVA/OA-1 and PVA/OA-3. In the case of the PVA/OA-4 was observed an increase in the value of the contact angle compared with PVA/OA-3. 

### 3.4. Thermal Stability

**Figure 7:**
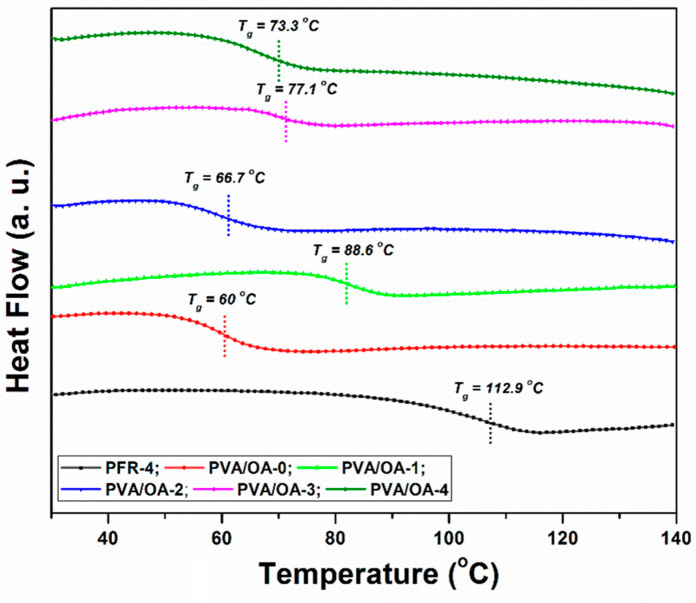
DSC curves for the PFR-4 and PVA/OA (0–4) films.

The differential scanning calorimetry (DSC) and thermal stability (TG) for the investigated PFR-4 and PVA/OA films were undertaken in order to investigate the physical status of PFR-4 and ze-Ag in the PVA/OA matrix. The DSC curves of PFR-4, original PVA/OA film, and PVA/OA- loaded with ze-Ag are displayed in Figure 7. As illustrated by DSC curves exhibited, the addition of PFR-4 obviously contributed to an increase in the T_g_, probably reflecting restricted internal rotation by the presence of DOPO groups. Compared with PVA/OA-0, the T_g_ value of PVA/OA-1 increased by approximately 28 °C. A decrease in T_g_ values could be observed in the case of the PVA/OA-2 compared with PVA/OA-1. Probably, the introduction of a larger amount of PFR-4 decreased the polymer chain interaction, thus decreasing the T_g_ values. The samples containing ze-Ag showed similar T_g_ values. The presence of the single T_g_ in the investigated samples can be ascribed to the good interfacial interaction between PVA/OA (1–4) and PFR-4, respectively Zeo or ze-Ag.

**Figure 8:**
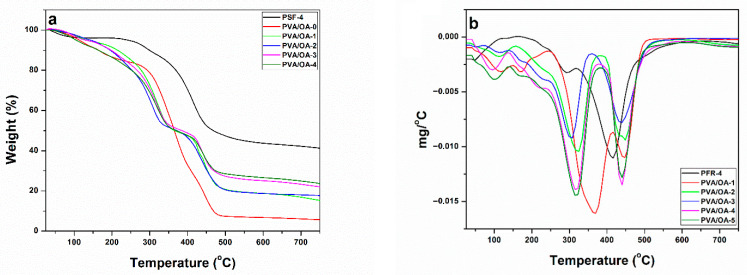
TG (**a**) and DTG (**b**) curves of the PFR-4, PVA/OA-0, and PVA/OA (1–4).

The TG and DTG curves of the investigated samples are presented in Figure 8a,b. The characteristics temperatures of thermal degradation including T_5%_, T_30%,_ and T_max_ as well as the char yields measured at 750 °C for these samples are summarized in Table 4. Compared with PVA/OA-0, the overall T_5%_ of the PVA/OA (1–4) is higher. In the case of the PVA/OA-4, the T_5%_ is lower probably the presence of the high content of ze-Ag in the sample leads to the catalysis of this. The weight loss of the PFR-4 took place between 267 °C and 361 °C, corresponding to the release of small molecules and breakdown of the weak bonds such as P–O–C and P–C [40,41,42,43,44,45]. Also, from the TG curves it can be observed that the PFR-4 can reduce the T_5%_ of the PVA/OA-0 and promote the formation of the carbonaceous layers on the surface. The char yield of PVA/OA-0 at 750 °C was 5.88%, while after the addition of PFR-4 and PFR-4/ze-Ag, the char yield of the samples increased from 15% to 24%, indicating improved flame retardancy. The DTG curves exhibit two steps of complete thermal decomposition. The first step can be attributed to the decomposition and carbonization of PVA/OA-0 chains, meanwhile, the second step might be caused by the decomposition of thermolabile fragments produced in the first step. The addition of PFR-4 yielded a slide decrease in the T_max_ of PVA/OA-0.

Using the data obtained from the TGA analysis the heat resistance index T_HRI_, which indicates the ability of polymers to resist a heat flow, can be calculated by using the following equation [46,47]:

T_HRI_ = 0.49[T_5%_ + 0.6(T_30%_ − T_5%_)]

Heat resistance index of the PFR, PVA/OA-0, and PVA/OA (1–4) was shown in Table 4. The highest values of T_HRI_ for the PFR, PVA/OA-0, and PVA/OA (1–4) were obtained for the sample denoted as PFR-4. Also, can be observed from Table 4 that the T_HRI_ decreased with the increase of the PFR-4 ratio into PVA/OA, but in the case of the samples containing PFR-4 and ze-Ag can be observed a reduction of these parameters.

### 3.5. Microscale Combustion Calorimetry (MCC) Tests

**Figure 9:**
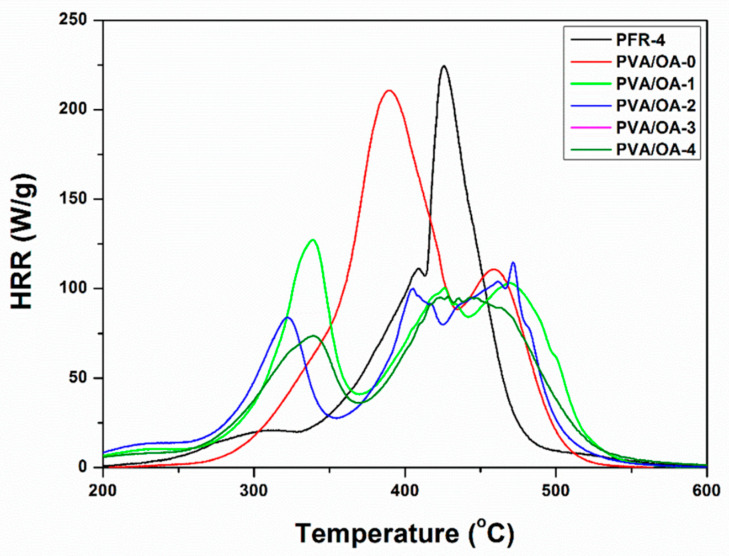
Heat release rates versus temperature for PFR-4 and PVA/OA films.

**Figure 10:**
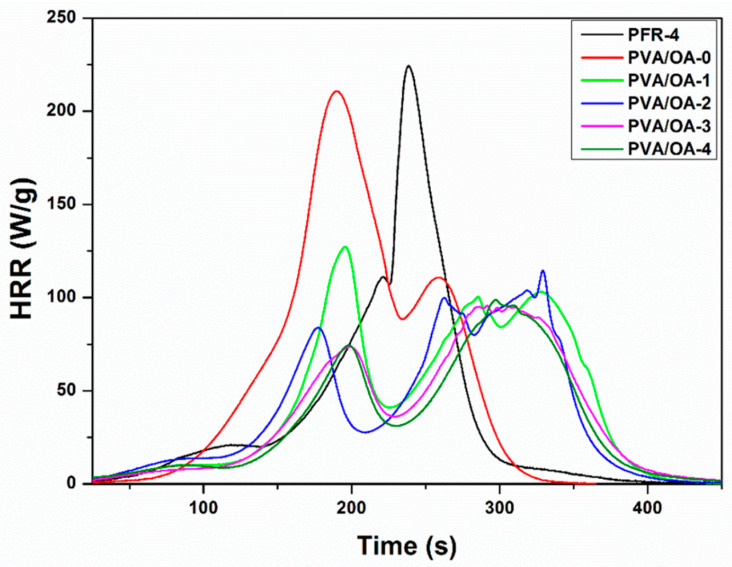
Heat release rates versus time for PFR-4 and PVA/OA films.

Microscale combustion calorimetry (MCC) tests were performed under controlled conditions. The most relevant data obtained from the MCC tests are presented in Table 5. In Figure 9 and Figure 10, the HRR curves were plotted, depending on temperature and time, respectively. In the case of the analyzed samples (PFR-4, PVA/OA (0–4)), a correlation is observed between HRC and Char Yield for the samples of compounds based on polyvinyl alcohol. Sample PVA/OA-0 has the highest HRC, 305.41 J/(g*K), and lowest Char Yield, 2.4%, and sample PVA/OA-4, with the lowest HRC of 164.57 J/(g*K) also has the highest Char Yield at 22.21%. There is thus an almost halving of 46.12% in HRC and an approximately 20% increase in Char Yield between sample PVA/OA-0 and sample PVA/OA-4. Polyvinyl alcohol samples with PFR-4 in the composition show a decrease in HRC of 21.5% for the PVA/OA-1 sample and 25.2% for the PVA/OA-2 sample, not even a 4% difference between the PVA/OA-1 sample and the PVA/OA-2 sample, which has double content of fire retardant. The addition of zeolite to the flame-retardant samples leads to a major decrease in the HRC, by about 20% compared to the fire retardant-only samples and by about 40% compared to the fire retardant-free sample, but without having large influences on the Char Yield and THR. However, excessive expansion of the silver-free zeolite sample is observed. The fire retardant of the ze-Ag samples (PVA/OA-3 and PVA/OA-4) shows the best fire behavior, from the point of view of the most important parameters discussed in the MCC analysis, both in relation to the PVA/OA-0 and in relation to the other fire retardant samples. It is possible that the addition of ze-Ag helps with the problem of expansion, but the phenomenon of bending of the samples occurs during the temperature rise. These samples, PVA/OA-3 and PVA/OA-4, thus record the lowest HRCs of about 165 J/(g*K), i.e., a decrease of 46% compared to the non-fire-retardant sample and a decrease of about 20% compared to the samples without ze-Ag. For these samples, the highest increase in Char Yield is also observed, but also the lowest decrease in THR, in relation to the non-fireproofed sample. Thus, the Char Yield increases by about 15% for sample PVA/OA-3 and about 20% for sample PVA/OA-4, and the THR decreases by about 23% and 30% for sample PVA/OA-3 and sample PVA/OA-4, respectively. These parameters, for the two samples with fire retardant and zeolite with silver, also show significant increases (Char Yield) and decreases (THR) compared to the fire retardant samples but without zeolite in the composition.

The fire retardant sample (PFR-4) was also analyzed, in a smaller amount than the other samples due to the high expansion potential. The PFR-4 has an HRC value close to that of the PVA/OA-0 sample, only 8% lower, but has almost the lowest THR and the highest Char Yield of 61.87% (Table 5). 

From the point of view of temperature (Figure 9), the increase in the temperature at which the PHRR is measured is observed for most of the fireproofed samples, except for sample PVA/OA-1, where the apparent temperature of the PHRR decreases by approximately 50 °C, compared to the non-fireproofed sample. Fire retardancy causes changes in the shape of the curves and the distribution of the peaks. Two prominent formations are observed in both the PVA/OA-0 sample and the fireproofed samples. Thus, the control sample shows two broad, close peaks, the first larger than the second. After fireproofing, a small release of heat is observed, followed by the first peak where, with the exception of sample PVA/OA-1, its narrowing and reduction are observed. Corresponding to the second peak of the control sample, for the flame-retardant samples the appearance of a broad formation is observed, without a very well-defined main peak.

Regarding the dependence on time (Figure 10), the delay in the appearance of the PHRR can be observed for all fireproofed samples, by up to 2 ± 0.3 min, the biggest delays being for samples PVA/OA-2, PVA/OA-3, and PVA/OA-4. The smallest delay occurs in sample PVA/OA-1, whose PHRR appears 0.11 min later than that of sample PVA/OA-0, which is also the sample with the first peak more prominent than the second peak formation. Analyzing the numerical data and the corresponding graphs of the results of the samples of compounds based on polyvinyl alcohol, it is observed that the samples with PFR-4 and ze-Ag show the best fire behavior, both in relation to the polyvinyl alcohol sample and in relation to the other samples fireproof. Polyvinyl alcohol samples with PFR-4 in the composition show a decrease in HRC of 21.5% for sample PVA/OA-1 and 25.2% for sample PVA/OA-2. The addition of zeolite to the flame retardant samples leads to a major decrease in the HRC, by about 20% compared to the fire retardant-only samples and by about 40% compared to the fire retardant-free sample, but without having large influences on the Char Yield and THR of the samples PVA/OA-3 and PVA-OA-4, thus record the lowest HRCs, of approximately 165 J/(g*K), i.e., a decrease of 46% compared to the non-fireproofed sample. For these samples, the highest increase in Char Yield is also observed, but also the lowest decrease in THR, in relation to the non-fireproofed sample. The addition of silver to these samples seems to solve the problem of expansion, but the phenomenon of bending occurs with the increase in temperature. Fireproofing is also successful in terms of temperature and timing of PHRR onset, with higher temperatures and later times being observed.

### 3.6. Mechanical Properties of Films

**Figure 11:**
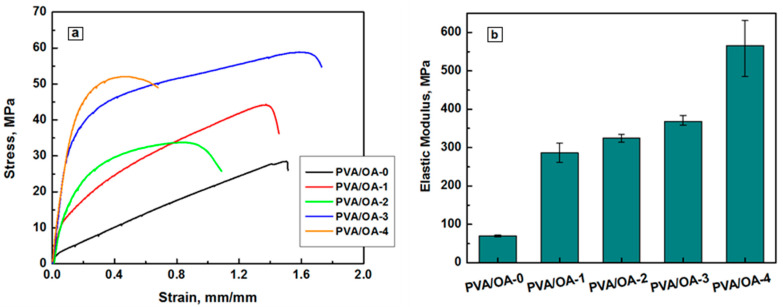
Stress-strain curves under tensile stress testing (**a**) and the representation of Young’s modulus (**b**) in the series.

Film-like materials are subjected to various external forces during practical uses. If the major mechanical properties fail to meet the basic requirements, their application fields will be largely restricted. Tensile strength and Young’s modulus are two key performance indexes for evaluating the mechanical properties of films. The mechanical behavior of the PVA/OA films reinforced with PFR-4 and ze-Ag is represented in Figure 11. The stress vs. strain curves of the PVA/OA films explicates the mechanical properties such as ultimate tensile strength (UTS), elastic modulus (Young’s modulus), and elongation at break (EB).

The UTS and YM of pure PVA/OA film were obtained at 27.46 ± 0.89 MPa and 70.10 ± 2.6 MPa, respectively. After incorporating the 0.2g PFR-4 in the blend matrix both the UTS and YM were significantly improved to 45.34 ± 1.33 MPa and 286 ± 25 MPa (Table 6).

### 3.7. Antimicrobial Properties

Testing by diffusion method of PVA/OA films showed the antimicrobial effect of zeolite/Ag embedded in PVA/OA-3 and PVA/OA-4 matrices (Table 7).

The PVA/OA films changed their size (10 mm) and even their original shape (disc), so for a more accurate interpretation of the results, the antimicrobial effect was evaluated by measuring the width of the microbial inhibition zone formed around the tested samples.

**Figure 12:**
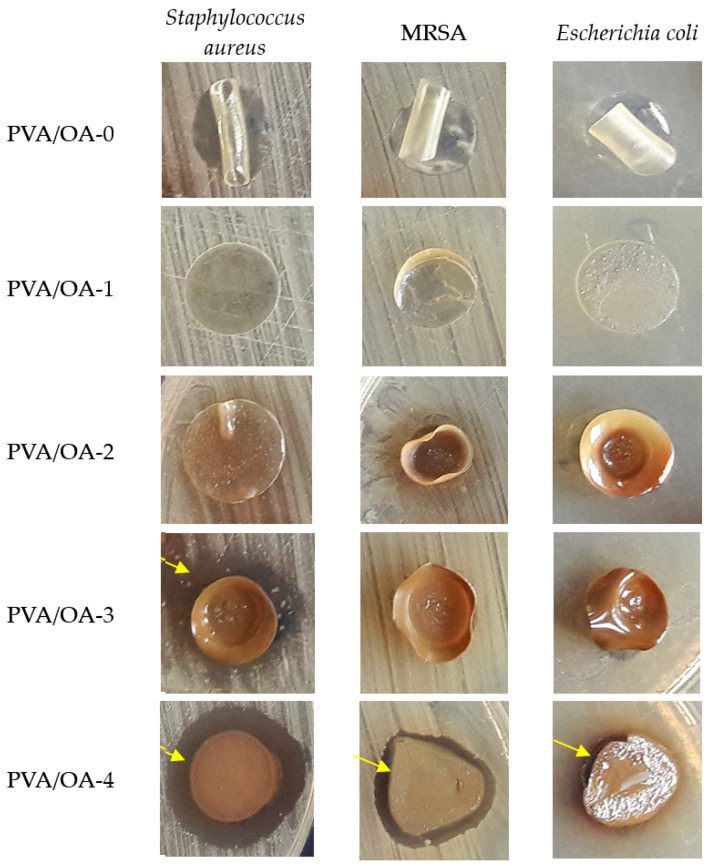
Aspects of antimicrobial activity of PVA/OA samples and areas of microbial inhibition.

PVA/OA-O, PVA/OA-1, and PVA/OA-2 samples did not cause microbial inhibition, while PVA/OA-3 determined variable zones of inhibition in *Staphylococcus aureus* species (4.66 ± 0.333 mm) and MRSA (1 mm). PVA/OA-4 films were the most active, showing significant antimicrobial activity against *Staphylococcus aureus* (5.66 ± 0.333 mm) and moderate activity against MRSA (2.66 ± 0.333 mm) and *Escherichia coli* (1.33 ± 0.333 mm), (Figure 12). The antimicrobial activity of the active samples was correlated and modeled with the presence of zeolite/Ag, as it was found that PVA/OA-3 (0.13 g) inhibited Gram-positive strains (*Staphylococcus aureus* and MRSA) and was not effective against Gram-negative strains (*Escherichia coli*), while doubling the amount of zeolite/Ag in PVA/OA-4 (0.26 g) enhanced antimicrobial activity against both groups of microorganisms, with the main role attributed to silver ions.

The effect of silver ions is significantly more effective against Gram-positive bacteria than against Gram-negative species. The differences in susceptibility may be due to the lower amount of negatively charged peptidoglycans in the cell walls of Gram-negative species [48].

Zeolites are a class of porous crystalline materials consisting of a three-dimensional framework of silicates and aluminates [49]. Their properties (biologically non-toxic, odorless, non-allergenic) make them safe for the environment and with positive effects on health [50,51].

Due to their porous structure, zeolites are good carriers for antimicrobial compounds and enhance antimicrobial activity by ion exchange. Silver is the most commonly used metal cation due to its recognized antimicrobial properties against a wide range of microorganisms [51,52,53].

The mechanism of action of zeolite L nanoparticles loaded with silver is based on the fact that they can release silver ions [51], penetrate the cell structure of microorganisms and disrupt their metabolic processes, ultimately leading to cell death [54,55].

Therefore, loading zeolite L nanoparticles with silver results in increased antimicrobial efficacy due to the combination of the antimicrobial properties of silver and the absorption and crosslinking properties of zeolite L. The results of our study support the research of other authors [52,54,56] who conclude that zeolite nanoparticles loaded with silver have a high potential for the development of materials and devices with antimicrobial activity due to their unique properties.

Overall, both zeolite and silver can be useful materials in packaging, particularly for products that are sensitive to moisture or that have a short shelf life. Zeolite is a porous material that can be used in packaging to absorb moisture and odors and to prevent the growth of bacteria and other microorganisms. This is particularly useful for products that are sensitive to moisture or that have a strong odor.

In our opinion, the presence of silver in combination with zeolite in the same packaging structure increases the quality of such finished products, and their use can extend the shelf life of food, followed by all the benefits that come from it.

## 4. Conclusions

The main objective of the current work was to develop innovative PVA-based composite films appealing for use in sustainable packaging applications by adjusting thermal properties, flame retardancy, and antimicrobial properties of the PVA via multiple material components and synergistic additive use. A phosphorus-containing bisphenol, PFR-4, has been utilized as an additive to impart flame retardancy while Ze-Ag nanoparticles were used to induce antimicrobial activity in the finite composites. Thermogravimetric analysis indicated that the introduction of PFR-4 and Ze-Ag nanoparticles increases the amount of residue at 700 °C. The MCC test indicated an improvement in flame resistance and a reduction in heat release capacity for samples containing phosphorus or phosphorus and Ze-Ag nanoparticles. Once loaded with silver, zeolite L nanoparticles are to be used as a highly effective antimicrobial agent due to the synergistic effect between the two materials. Silver ions can diffuse out of the zeolite L nanoparticles, attacking microbes by binding to their cell membranes and disrupting cellular function. At the same time, the porous structure of the zeolite L nanoparticles provides a unique surface area for the absorption of organic molecules, further enhancing the antimicrobial efficacy of the material. Loading zeolite L nanoparticles with silver is a promising approach for developing new antimicrobial agents with enhanced efficacy and performance.

## Figures and Tables

**Table 1:** Preparation of the PVA/OA films.

Sample	PVA (g)	Oxalic Acid (g)	PFR-4 (g)	Ze-Ag (g)
PVA/OA-0	1.70	0.30	-	-
PVA/OA-1	1.53	0.27	0.20	-
PVA/OA-2	1.36	0.24	0.40	-
PVA/OA-3	1.42	0.25	0.20	0.13
PVA/OA-4	1.31	0.23	0.20	0.26

**Table 2:** Contact angles of distilled water and ethylene glycol (EG) for the investigated films.

Sample	Contact Angles (°)				
	PVA/OA-0	PVA/OA-1	PVA/OA-2	PVA/OA-3	PVA/OA-4
Water	131.49 ± 0.28	99.41 ± 1.02	114.63 ± 1.95	113.04 ± 0.92	124.66 ± 0.87
EG	94.38 ± 0.96	73.69 ± 1.12	62.66 ± 0.47	70.57 ± 0.56	63.85 ± 0.49

**Table 3:** Adhesion work and the surface tension for the PVA/OA films.

Sample	W	γ_SV_^p^	γ_SV_^d^	γ_SV_	γ_SL_
PVA/OA-0	24.6/44.3	4.25	33.5	37.7	27.04
PVA/OA-1	60.9/61.5	1.27	22.9	24.3	36.2
PVA/OA-2	42.5/70.1	7.38	75.75	83.1	35.8
PVA/OA-3	44.3/63.9	2.75	53.03	55.8	36.9
PVA/OA-4	31.4/69.1	18.04	97.2	115.2	35.3

**Table 4:** TG and DTG data of the PFR-4, PVA/OA-0 and PVA/OA (1–4).

Samples	T_5%_ ^a^ (°C)	T_30%_ ^b^ (°C)	T_HRI_ ^c^ (°C)	T_max_ ^d^ (°C)	Residue at 750 °C
PFR-4	244	401	165.72	294; 415	42
PVA/OA-0	119	323	118.29	371; 446	6
PVA/OA-1	131	308	116.23	324; 448	15
PVA/OA-2	140	290	112.7	305; 435	18
PVA/OA-3	127	307	115.15	220; 319; 436	22
PVA/OA-4	103	299	108.09	319; 436	24

^a^ temperature where 5 wt% of the weight was lost; ^b^ decomposition temperature of 30% weight loss; ^c^ heat resistance index; ^d^ the first DTG peak.

**Table 5:** Data obtained by MCC analysis for the PFR-4 and PVA/OA films.

Samples	Weight (mg)	Char Yield (mg)	Char Yield (wt%)	Decomposition Rate (%)	HRC (J/(g*K))	THR (kJ/g)	PHRR (W/g)	T_PHRR_ (°C)	Time (s)
PFR-4	10.61	4.30	61.87	38.13	280.18	15.18	224.37	425.75	238.50
PVA/OA-0	20.01	0.49	2.45	97.55	305.41	20.77	210.80	389.17	189.50
PVA/OA-1	20.02	2.03	10.14	89.86	239.84	18.66	127.14	339.42	196.00
PVA/OA-2	20.01	2.97	14.84	85.16	228.33	16.66	114.69	471.47	329.00
PVA/OA-3	19.96	3.39	16.98	83.02	165.66	16.02	95.70	428.64	291.50
PVA/OA-4	19.95	4.43	22.21	77.79	164.57	14.63	98.91	442.76	297.50

CY = char yield; PHRR = Heat release peak; THR = Total heat release; T_PHRR_ = Temperature of heat release peak; Time = The time to attain heat release peak; HRC = Heat release capacity.

**Table 6:** Data obtained by mechanical testing of the film composites.

Sample	Young’s Modulus, MPa	Maximum Tensile Stress (MPa)	Elongation at Break (mm/mm)
PVA/OA-0	70.10 ± 2.36	27.46 ± 0.89	1.36 ± 0.15
PVA/OA-1	286.28 ± 25	45.34 ± 1.34	1.61 ± 0.23
PVA/OA-2	324.73 ± 10	33.71 ± 0.5	0.87 ± 0.01
PVA/OA-3	368.53 ± 15	54.08 ± 4.6	1.33 ± 0.31
PVA/OA-4	565.64 ± 65	52.06 ± 2.5	0.47 ± 0.02

**Table 7:** The mean value and standard deviation (SD) of the inhibition zones were obtained to antimicrobial testing against bacteria strains.

Samples Ø 10 mm	Inhibition Zone (mm)		
	*Staphylococcus aureus* ATCC 25923	*Methicillin-Resistant Staphylococcus aureus* (MRSA) ATCC *33591*	*Escherichia coli* ATCC 25922
	Mean ± SD	Mean ± SD	Mean ± SD
PVA/OA-0	0	0	0
PVA/OA-1	0	0	0
PVA/OA-2	0	0	0
PVA/OA-3	4.66 ± 0.333	1	0
PVA/OA-4	5.66 ± 0.333	2.66 ± 0.333	1.33 ± 0.333

## Data Availability

The data that support the findings of the current study are listed within the article.
